# Baseline coral disease surveys within three marine parks in Sabah, Borneo

**DOI:** 10.7717/peerj.1391

**Published:** 2015-11-03

**Authors:** Jennifer Miller, Michael J. Sweet, Elizabeth Wood, John Bythell

**Affiliations:** 1 School of Biology, Newcastle University, Newcastle upon Tyne, UK; 2 Marine Conservation Society, Ross-On-Wye, UK; 3 Semporna Islands Darwin Project, Tun Sakaran Marine Park Complex, Malaysia

**Keywords:** Coral disease, Marine park, White syndrome, Coral cover, Bleaching, Atramentous necrosis, Borneo

## Abstract

Two of the most significant threats to coral reefs worldwide are bleaching and disease. However, there has been a scarcity of research on coral disease in South-East Asia, despite the high biodiversity and the strong dependence of local communities on the reefs in the region. This study provides baseline data on coral disease frequencies within three national parks in Sabah, Borneo, which exhibit different levels of human impacts and management histories. High mean coral cover (55%) and variable disease frequency (mean 0.25 diseased colonies m^−2^) were found across the three sites. Highest disease frequency (0.44 diseased colonies per m^2^) was seen at the site closest to coastal population centres. Bleaching and pigmentation responses were actually higher at Sipadan, the more remote, offshore site, whereas none of the other coral diseases detected in the other two parks were detected in Sipadan. Results of this study offer a baseline dataset of disease in these parks and indicate the need for continued monitoring, and suggest that coral colonies in parks under higher anthropogenic stressors and with lower coral cover may be more susceptible to contracting disease.

## Introduction

Tropical marine environments are faced with unprecedented natural and anthropogenic disturbances (Doney, 2006; Pratchett, Hoey & Wilson, 2014). Even optimistic scenarios for environmental change suggest mass declines in coral cover and species richness (Hoegh-Guldberg et al., 2007; Veron et al., 2009; Fabricius et al., 2011). It has been predicted that these ecosystems, which are vital to the economy and subsistence of approximately 500 million people, could be lost within the next century (Wilkinson, 2008; Brierley & Kingsford, 2009). The biggest threats to the longevity of coral reefs are often reported as being bleaching events and outbreaks of disease (Knowlton, 2001; Harvell et al., 2002; Gardner et al., 2003; Baker et al., 2004). Coral disease can lead to significant decreases in live coral cover (Miller et al., 2009), partial to whole-colony mortality (Willis, Page & Dinsdale, 2004) and changes in community composition (Bythell & Sheppard, 1993; Francini-Filho et al., 2008) consequently jeopardising the entire reef ecosystem (Bellwood et al., 2004; Sweet, Croquer & Bythel, 2014).

Disease outbreaks have been temporally and spatially linked to bleaching events (Muller et al., 2008; Bruckner & Hill, 2009; Croquer & Weil, 2009), and sea surface temperature (SST) anomalies have been correlated with increases in bleaching and disease prevalence (Bruno et al., 2007; Heron et al., 2010; Ruiz-Morenol et al., 2012). Current SST levels are at their highest on record (NOAA, 2006) and are predicted to increase in the future, particularly in the tropics (Hoegh-Guldberg et al., 2007) so the number of bleaching and disease outbreaks is also likely to increase. Additionally, increased coastal populations of humans and the associated anthropogenic stressors such as pathogen transportation (Harvell et al., 1999) pollution and eutrophication (Bruno et al., 2003), fishing and dredging (Nyström, Folke & Moberg, 2000) may increase coral susceptibility to diseases (Bourne et al., 2015).

The number of coral diseases identified worldwide varies between studies, ranging from 5 to 29 (Dalton & Smith, 2006), with 22 reported in the Atlantic (Weil, 2004) and 11 in the Indo-Pacific (Global Coral Disease Database; http://coraldisease.org/, accessed January 2015). There has been extensive research on the microbial pathogens associated with a variety of coral diseases; however the application of Koch’s postulates (Koch, 1982) is problematic in the marine environment. This may be partly due to the difficulty in confidently separating stress responses from signs of disease in corals under laboratory conditions and also the difficulty in defining coral disease signs in the laboratory in relation to those in the field. Therefore, causal agents of few coral diseases have been identified and are increasingly disputed (Harvell et al., 2007). Indo-Pacific reefs have almost ten times higher scleractinian species richness and coral cover than Caribbean reefs (Bruno & Selig, 2007). However, a lower disease prevalence and slower decline in coral cover is reported in the Indo-Pacific in most (Goldberg, Wilkinson & Wilkinson, 2004; Willis, Page & Dinsdale, 2004) but not all studies (Dinsdale, 2000). This difference could be explained by variation in coral community species composition and susceptibilities to diseases (Connell, 1997; Sutherland, Porter & Torres, 2004). Alternatively, a paucity of disease studies in the Indo-Pacific (Goldberg, Wilkinson & Wilkinson, 2004; Wilkinson, 2008; Harvell et al., 2007) and a focus on epizootics in the Caribbean (e.g., Croquer, Pauls & Zubillaga, 2003) may represent a significant bias.

Despite the biological and economic significance of coral reefs in the south-east Asian region, no systematic investigation of coral diseases has been conducted in Malaysia. Land clearance and future development plans for rapid increases in population sizes on the Malaysian coast (Comley et al., 2004) will augment pressures on marine ecosystems. This is especially so when mangrove and other coastline vegetation has been lost or degraded, removing natural barriers to coastal erosion and consequent land runoff (Wilkinson, 2008). Sabah in Borneo supports around 550 (Burke & Selig, 2002a; Burke & Selig, 2002b) of the Indo-Pacific’s 581 scleractinian coral species (Veron, 2000) and represents 75% of Malaysia’s coral reef area (WRI, 2010). However, these reefs are also included in the world’s 15% most critically threatened reefs (Wilkinson, 2008) and in 2011 97% of Malaysia’s reefs were categorised as threatened, and 50% as under high threat of severe degradation (Burke & Selig, 2002a; Burke & Selig, 2002b; Burke et al., 2011). The present study establishes the first baseline coral disease data-set within three national parks in Sabah, Malaysian Borneo, and therefore improves the geographical distribution of data of coral disease frequency in tropical reef environments.

## Materials and Methods

### Survey area

**Figure 1:**
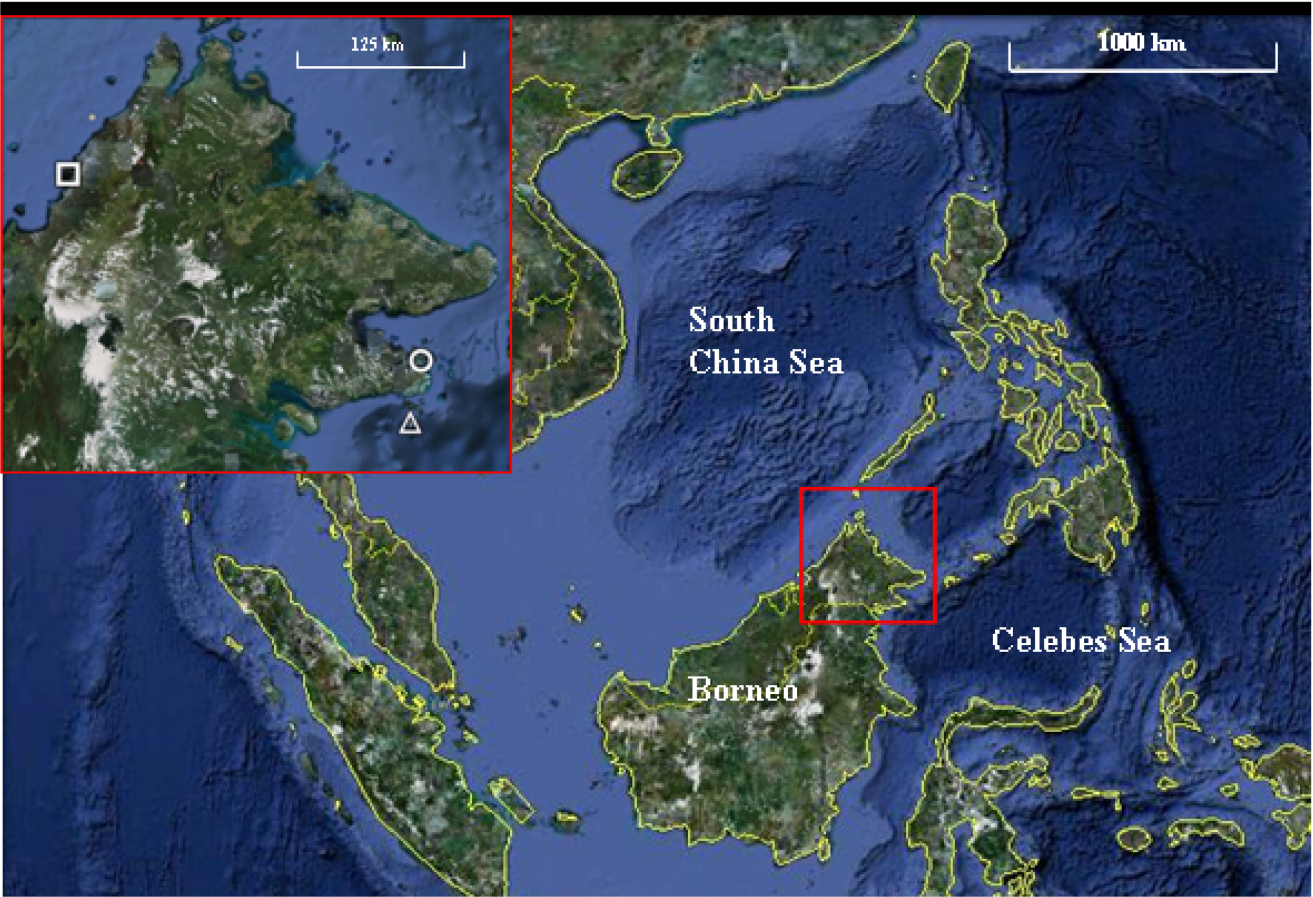
Map of the three marine parks surveyed. Tunku Abdul Rahman Park (TARP), Tun Sakaran Marine Park (TSMP) and Sipadan Island Park (SIP) in Sabah, Malaysian Borneo (inset) were surveyed May–June 2010. TARP (Inset, circle; grid references 6°02′N, 116°05′E) is a 50 km^2^ park in the south China Sea, 3 km from west Sabah’s coastline. TSMP (Inset, triangle; grid references 4°42′N, 118°51′E) is a 350 km^2^ park in the Celebes Sea, 10 km from the east Sabah’s coastline. SIP (Inset, square; grid references 4°7′N, 118°37.5′E) is a protected island 35 km south-east from Semporna, Sabah. Grid references were recorded at each transect site using Garmin GPS Map 76 handheld mapping equipment. Map data: Google Earth, DigitalGlobe. Scale bar = 1,000 km. Inset scale bar = 125 km.

The study was conducted at three parks, Tunku Abdul Rahman Park (TARP), Tun Sakaran Marine Park (TSMP) and Sipadan Island Park (SIP) in Sabah, Malaysian Borneo (Fig. 1). All surveys were conducted between May and June 2010. The three parks were chosen for their varying levels of disturbance due to their proximity to the mainland and levels of fishing permitted. TARP covers 50 km^2^ area but is only 3 km from Kota Kinabalu (the capital city of Sabah) in the South China Sea (Fig. 1) and has had protected status since 1977. Indigenous communities of fishing villagers reside within the park boundaries and hook and line fishing is permitted throughout. TSMP covers 350 km^2^ area, is 10 km from the nearest city—Semporna (on the east coast of Sabah), and was gazetted in 2004 (Wood, 2001a; Wood, 2001b). Hook and line fishing is permitted in specific zones, and the park has its own enforcement staff to regulate this. SIP is a small island isolated by deep-water channels, 35 km from Semporna, covering an area of 168 km^2^ of water and reef. SIP has been partially protected since 1930 and fully gazetted since 1963. SIP remains a complete no take zone, enforced by the Sabah Parks National Park Authority. Access was permitted by the Economic Planning Unit of Malaysia (permit ID:TS/TMTS/UPM/P/4/4[48]).

### Disease surveys

At each of the 24 randomly selected survey sites, one 100 × 0.5 m was surveyed for occurrence of coral diseases and % hard coral cover. 8 transects were surveyed at TARP, TSMP and SIP 4 at 10 m and 4 at 5 m depth. In total an area of 1,200 m^2^ was surveyed. All cases of coral disease, bleaching and pigmentation response, affecting colonies in the transect area wereafter identified and categorised according to Coral Reef Targeted Research—Disease Working Group guidelines (Beeden et al., 2008, Table 1). Close-up photographs (using Canon Powershot G11 cameras), were taken of each disease enabling later verification and standardisation of diseaseidentification. Percent hard coral cover was recorded during the surveys and was later verified using examination of high resolution photographs taken at 0, 50 and 100 m distance points along the transect, approximately 2–3 m above the transect depth. Survey sites were selected using a random number generator choosing from possible GPS coordinates.

**Table 1:** Coral diseases observed and the identification methods used in the present study. All known diseases as described in the Coral Reef Targeted Research Disease Working Group’s guidelines for “Assessing Coral Health on Indo-Pacific Reefs” guidelines were included in the surveys but only those listed in Table 1 were observed.

Disease/syndrome	Notes for identification	References for identification methods
Atramentous Necrosis (AtN)	Patchy bleaching, distinct grey-blue microbial “mats” covering necrotic tissue lesions	Willis, Page & Dinsdale (2004) and Beeden et al. (2008)
Black Band Disease (BBD)	Distinctive dark band separating live tissue and exposed skeleton & relatively quick progression	Beeden et al. (2008), Miller (1996) and Richardson (2004)
Bleaching (BL)	Translucent tissue revealing white skeleton underneath & may be patchy, affecting whole colony or communities	Beeden et al. (2008)
Brown Band Disease (BrB)	Brown band of “active” disease delineating live and dead/absent tissue, sometimes bands of bleached tissue between disease and live tissue, rapid progression	Bourne et al. (2008) and Beeden et al. (2008)
Pigmentation Response (PR)	Fluorescent pink/purple/blue patches or spots, particularly around skeleton borers, parasites and algal overgrowth, unlikely mortality	Willis, Page & Dinsdale (2004) and Beeden et al. (2008)
White Syndrome (WS)	Distinct boundary of white–yellow (with time) exposed skeleton and live tissue, some bleached tissue may occur between live and diseased areas, rapidly progresses	Willis, Page & Dinsdale (2004), Bythell, Pantos & Richardson (2004) and Beeden et al. (2008)
Predation	Scarring from corallivorous fish, crown of thorns etc. leaving tissue loss and skeleton abrasion	Raymundo et al. (2008)

### Data analysis

The frequency of disease, bleaching and pigmentation response was calculated per unit area of surveyed reef (i.e., colonies affected per m^2^) for each transect before statistical analysis. The difference between the two depths of survey transects was tested with a nested *t*-test. Data from categories’ disease + bleaching + pigmentation response’, plus ‘bleaching’ and ‘pigmentation response’ alone satisfied a normal distribution so a nested site within park Analysis of Co-Variance (ANCOVA) was carried out to test for differences in frequency of these three categories between the three parks, with coral cover being a covariate. Additionally, the Kruskal–Wallis test was used to analyse differences in overall frequencies of all diseases, WS and AtN (these data did not satisfy a normal distribution) between TARP and TSMP. A linear regression was also used to investigate the correlation between % coral cover and disease frequencies and between bleaching and disease frequencies in TARP and TSMP. All analyses were carried out using Minitab 17 software, except the linear regression which was carried out in R using the MASS package (Venables & Ripley, 2002).

## Results

### Disease frequency

**Figure 2:**
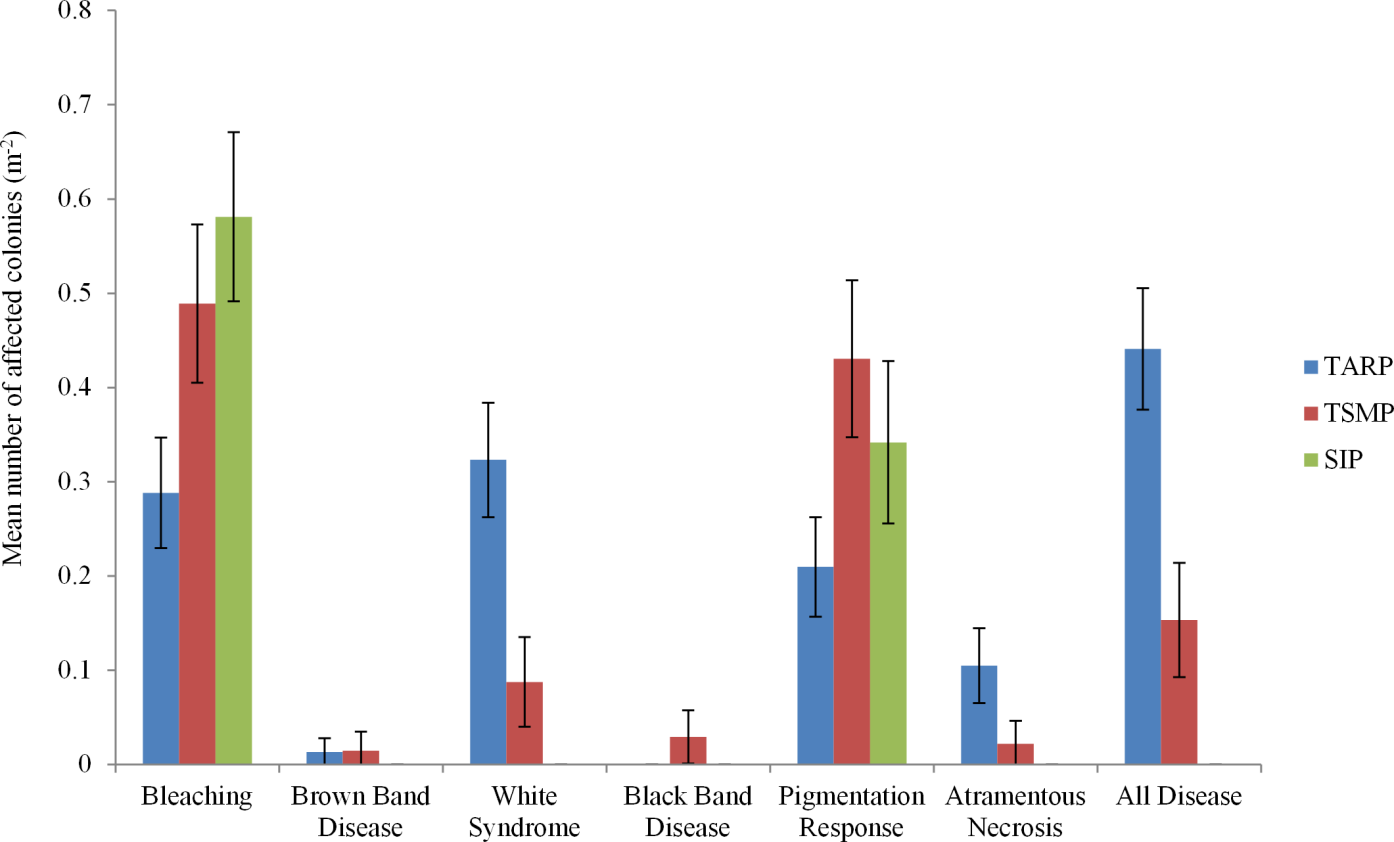
The frequencies of all diseases, bleaching and pigmentation response (m^−2^) recorded at the three marine parks surveyed. Tunku Abdul Rahman Park, TARP; Tun Sakaran Marine Park, TSMP and Sipadan Island Park, SIP; Disease, frequency of all recorded diseases (categories ‘White Syndrome,’ ‘Brown Band,’ ’Black Band,’ and ‘Atramentous Necrosis’). ±95% confidence intervals.

Three diseases commonly found in the Indo-Pacific region were not observed in Sabah during the present surveys: Skeletal Eroding Band, *Porites* Ulcerative White Spot and *Porites* Trematodiasis. There was no significant difference between the disease frequencies at 10 and 5 m depth at any park. A total of 443 colonies were recorded showing indications of immune responses or disease, including 95 with signs of a recognised coral disease (Atramentous Necrosis (AtN), Black Band Disease (BBD), Brown Band Disease (BrB) or White Syndrome (WS)), 201 partially or fully bleached colonies and 147 showing signs of a Pigmentation Response (PR) (Table 2). Diseases, bleaching and pigmentation responses pooled differed significantly between the three marine parks (nested ANCOVA: *F* = 7.08, *P* = 0.005, Table 2), and there was high variation in different frequencies of the different diseases observed (Fig. 2). The frequency of coral disease (excluding bleaching and pigmentation responses) was significantly higher at TARP than TSMP (Kruskal–Wallis: *H* = 20.23, *P* = < 0.0001), and none of the coral diseases widely recognised were observed at SIP (Table 2). WS was the most commonly observed disease, with a total of 74 and 12 colonies at TARP and TSMP respectively. The frequency of WS and AtN were both significantly higher at TARP than TSMP (Kruskal–Wallis: *H* = 18.6, *P* = < 0.001 and *H* = 11.09, *P* = < 0.001 respectively). Two other commonly observed diseases in the Indo-Pacific, Brown Band Disease (BrB) and Black Band Disease (BBD) were recorded during the surveys but at low frequencies, with BrB present at both TARP and TSMP, and BBD was only observed at TSMP. Bleaching and pigmentation responses were more frequent at SIP, although these differences were not significant, and bleaching was not significantly correlated with disease frequency at either TSMP or TARP.

**Figure 3:**
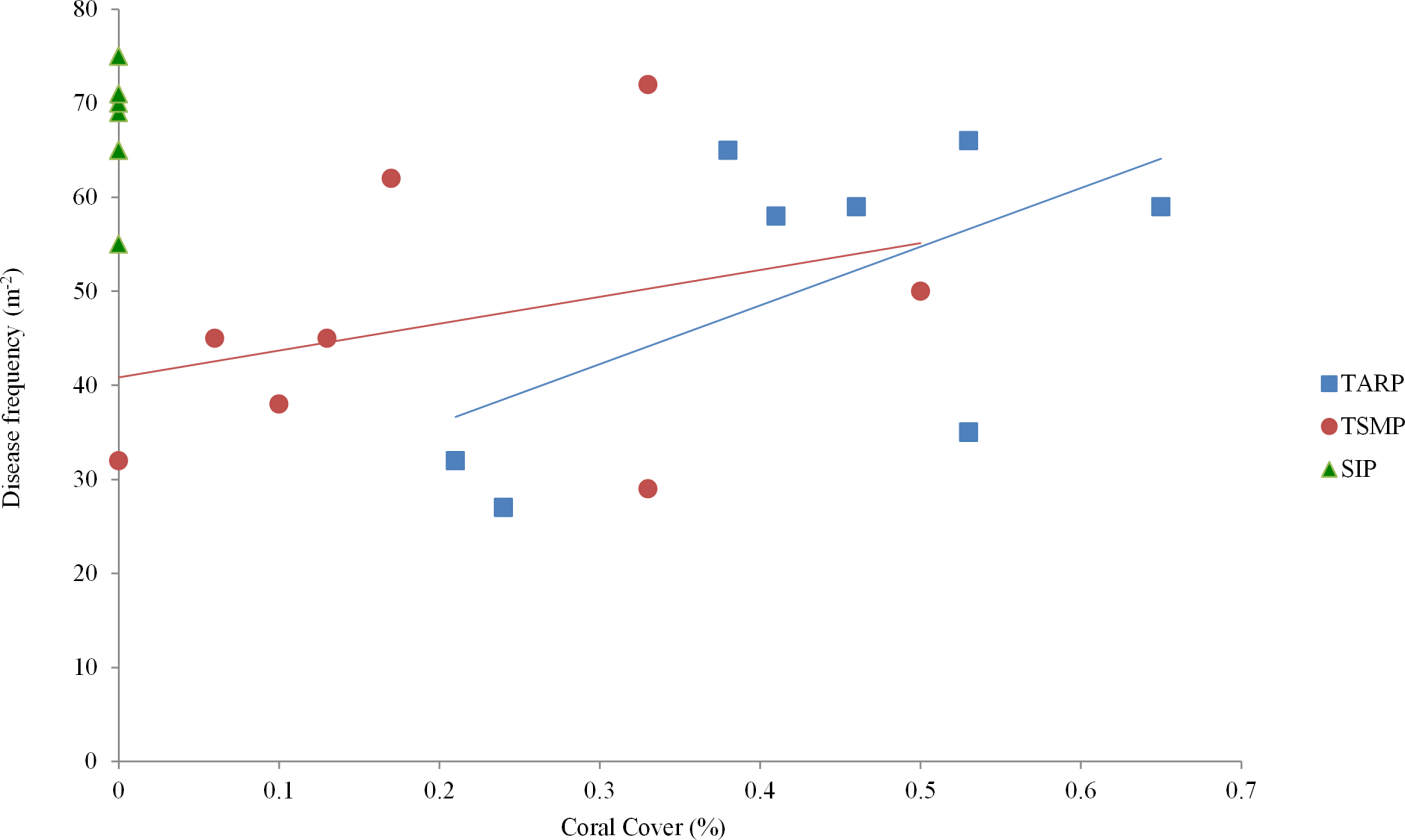
The relationship between percent coral cover and disease frequency at three parks surveyed. Tunku Abdul Rahman Park, TARP; Tun Sakaran Marine Park, TSMP and Sipadan Island Park, SIP. A positive correlation was found between coral cover and disease frequency at TARP (Linear regression: *r*^2^ = 0.48, *p* < 0.01) but not at TSMP (Linear regression: *r*^2^ = 0.57, *p* > 0.05). No disease was observed at SIP.

**Table 2:** Mean frequency (no. coral colonies affected per **m^2^**) of each coral exhibiting disease, bleaching and pigmentation response. Three marine parks were surveyed in Sabah, Borneo. Where significantly different frequencies were recorded, the value is followed by a suffix (∗ or +) not shared by other values in that column.

Park	Disease	Bleaching	WS	BrB	BBD	AtN	PR
TARP	0.44*	0.28	0.32*	0.01*	0	0.10*	0.21
TSMP	0.15^+^	0.49	0.09^+^	0.01*	0.02	0.02	0.43
SIP	0	0.58	0	0	0	0	0.34
Mean	0.25	0.42	0.18	0.01	0.008	0.06	0.30

**Notes.**

TARP Tunku Abdul Rahman Park TSMP Tun Sakaran Marine Park SIP Sipadan Island Park

Column headings Disease All diseases WS White syndrome BrB Brown band disease BBD Black Band Disease AtN Atramentous Necrosis PR Pigmentation

### Coral cover

Mean coral cover in TARP, TSMP and SIP was 50.1 ± 14.9%, 46.6 ± 13.7% and 68.1 ± 6.1% respectively (mean ± s.d.), with no significant difference between the three park sites in the relationship of coral cover and frequency of all diseases. A positive relationship was found between coral cover and disease at TARP (*r*^2^ = 0.48, *p* < 0.01, Fig. 3) but not at TSMP.

## Discussion

### Disease variation

White Syndrome (WS) was observed at the highest frequency of all diseases recorded, especially at TARP, where the number of colonies per m^2^ was four times higher than at TSMP. Some mis-categorisation of observed WS-like signs may explain some of this variance as predation, bleaching, white spot diseases, atramentous necrosis, brown band disease and skeletal eroding band are often mistaken for WS during rapid disease surveys (Willis, Page & Dinsdale, 2004; Lindop, Hind & Bythell, 2009). However, such significant differences between parks is unlikely to be explained by erroneous identification alone, given that the same observers conducted all surveys, photographs of diseased colonies were verified by one researcher after each survey and no diseases were observed at SIP.

The frequency of corals with bleaching and pigmentation response (PR) was higher in SIP than at either TARP or TSMP, although the differences were not significant. PR is thought to be an indicator of immune function in corals through the expression of a fluorescent protein linked to immune responses (Palmer, Mydlarz & Willis, 2008; Palmer, Roth & Gates, 2009), and frequently results from parasite infections and predation scars (Willis, Page & Dinsdale, 2004; Aeby, 2007). The higher PR frequency observed in SIP could therefore be explained by a greater predation on corals by fishes, whose populations are promoted due to the more effective park protection at SIP, possibly due to logistics of policing such a small park area. In comparison, despite being gazetted for over 30 years, there are still local fishing villages situated within TARP boundaries and illegal blast fishing and poaching does continue (particularly at the park edges and borders), in addition to the permitted hook-and-line fishing throughout the park (Wood, 2001a; Wood, 2001b). TSMP was established in 2005, and so relatively recently in terms of the reduction of fishing pressure. However, no fishing has been permitted, under strict enforcement in SIP, since the island became protected in 1963. The presence of the White Syndrome at TARP and TSMP is more concerning than the occurrence of Pigmentation Response, due to the recognized mortality associated with White Syndrome (Willis, Page & Dinsdale, 2004; Bourne et al., 2015). Although in the present study we did not have resources to carry out more than one survey, in future, surveys should be repeated to verify pigmentation responses observed is not a precursor for white syndrome or other disease.

### Coral cover relationships

Coral cover and disease was positively correlated only at TARP. Some previous studies that have surveyed coral disease on a frequency per unit reef area basis did not record coral cover during field surveys (e.g., Haapkyl et al., 2007) whilst others did not test the relationship of coral cover and disease prevalence, either comparatively between surveyed sites or as an overall correlation (e.g., Kaczmarsky, 2006; Bruckner & Hill, 2009). Without such analyses, it is difficult to compare between studies and between sites/times since possible variations in host density are unknown. The variation in the relationship between host density (coral cover), and disease frequency has been shown in this study to be a useful indicator in itself. Higher disease frequency per unit coral cover at TARP indicates that factors other than host density are influencing the presence of coral disease at this park.

### Inter-park environmental variance

Human disturbances, such as habitat fragmentation, pollution and sedimentation, are predicted to reduce reef resilience to natural stressors (Wilkinson, Souter & Goldberg, 2006), which subsequently may reduce the immune defence capacity of the coral holobiont against diseases (Reshef et al., 2006; Ritchie, 2006). Sedimentation and nutrient-rich effluent from intensive coastal-based agriculture (especially around the capital city of Sabah, Kota Kinabalu (Jakobsen et al., 2007)) just 3 km from TARP) and turbidity levels as high as 39.3 Nephelometric Turbidity Units (Anton et al., 2008), may explain the higher disease frequency observed at TARP compared to the other two sites. In addition to indirect stress effects, there is a potential for direct introduction of coral disease pathogens via human effluent, which has been recorded to be discharged into Kota Kinabalu’s rivers and therefore estuaries (Sakari et al., 2012), and has recently been associated with the onset of Caribbean White Spot disease in Elkhorn coral (Sutherland et al., 2010). Kaczmarsky, Draud & Williams (2005) also recorded around ten times higher disease prevalence of corals in waters in the US Virgin Islands with a sewage pollution indicator microbe species compared to those without (Kaczmarsky, Draud & Williams, 2005). The lack of an efficient sewage filtration system at Kota Kinabalu (Jakobsen et al., 2007) may therefore directly affect coral disease prevalence at sites close by. However, further investigation into the links between coral disease with quantified gradients of relevant pollutants, contaminants or indicator microbes, would need to be carried out to verify whether or not such influences are occurring in Sabah. Additionally, the more open access to TARP and lower ratio of staff to park area to police tourism and resident activity may increase damage to reefs from SCUBA divers, boat anchoring and other activities which have been linked to increased disease prevalence (Lamb et al., 2014), whereas SCUBA diving and boat anchoring is strictly regulated and controlled in SIP.

In conclusion, there were significantly greater coral disease frequencies in TARP, particularly WS and AtN compared to the other two sites, TSMP and SIP. The relationships between disease frequencies and coral cover was found to be different at TARP from TSMP and SIP inferring that inter-park variance in disease may be due to factors other than host density. The proximity of TARP to the developing coastline of Kota Kinabalu and associated anthropogenic pressures such as reduced water quality, along with high numbers of tourist and illegal fishing activity appear the most likely explanation for increased disease frequency at this site, but further research into these influences are needed. The apparent absence of recognised diseases at the most effectively protected, offshore site (SIP), is encouraging and the results suggest that bleaching and pigmentation responses are not necessarily indicative of overall less healthy reefs.

## Supplemental Information

10.7717/peerj.1391/supp-1 Supplemental Information 1 Frequency of diseases and health indicators

## Additional Information and Declarations

### Field Study Permissions

The following information was supplied relating to field study approvals (i.e., approving body and any reference numbers): The Research Promotion and Co-ordinate Committee (Malaysian Economic Planning Unit) issued a letter stating approval of our planned research prior to its start, following an application submitted by the primary author (Permit ID:TS/TMTS/UPM/P/4/4[48]).

## References

[ref-1] Aeby GS (2007). Spatial and temporal patterns of Porites trematodiasis on the reefs of Kaneohe Bay, Oahu, Hawaii. Bulletin of Marine Science.

[ref-2] Anton A, Teoh PL, Mohd-Shaleh SR, Mohammad-Noor N (2008). First occurrence of Cochlodinium blooms in Sabah, Malaysia. Harmful Algae.

[ref-3] Baker AC, Starger CJ, McClanahan TR, Glynn PW (2004). Coral reefs: corals’ adaptive response to climate change. Nature.

[ref-4] Beeden R, Willis RL, Raymundo LJ, Page CA, Weil E (2008). Underwater cards for assessing coral health on Indo-Pacific reefs.

[ref-5] Bellwood DR, Hughes TP, Folke C, Nystrom M (2004). Confronting the coral reef crisis. Nature.

[ref-6] Bourne DG, Ainsworth TD, Pollock FJ, Willis BL (2015). Towards a better understanding of white syndromes and their causes on Indo-Pacific coral reefs. Coral Reefs.

[ref-7] Bourne DG, Boyett HV, Henderson ME, Muirhead A, Willis BL (2008). Identification of a ciliate (Oligohymenophorea: Scuticociliatia) associated with brown band disease on corals of the Great Barrier Reef. Applied and Environmental Microbiology.

[ref-8] Brierley AS, Kingsford MJ (2009). Impacts of climate change on marine organisms and ecosystems. Current Biology.

[ref-9] Bruckner AW, Hill RL (2009). Ten years of change to coral communities off Mona and Desecheo Islands, Puerto Rico, from disease and bleaching. Diseases of Aquatic Organisms.

[ref-10] Bruno JF, Petes LE, Harvell DC, Hettinger A (2003). Nutrient enrichment can increase the severity of coral diseases. Ecology Letters.

[ref-11] Bruno JF, Selig ER (2007). Regional decline of coral cover in the Indo-Pacific: timing, extent and subregional comparisons. PLoS ONE.

[ref-12] Bruno JF, Selig ER, Casey KS, Page CA, Willis BL, Harvell CD, Sweatman H, Melendy AM (2007). Thermal stress and coral cover as drivers of coral disease outbreaks. PLoS Biology.

[ref-13] Burke L, Reytar K, Spalding M, Perry A (2011). Reefs at risk revisited.

[ref-14] Burke L, Selig ER, Institute WR (2002a). Reef density in Sabah. Reefs at risk in southeast Asia.

[ref-15] Burke L, Selig ER (2002b). Reefs at risk in south east Asia.

[ref-16] Bythell J, Pantos O, Richardson L, Rosenberg E, Loya Y (2004). White plague, white band and other “white” diseases. Coral health and disease.

[ref-17] Bythell J, Sheppard C (1993). Mass mortality of Caribean shallow corals. Marine Pollution Bulletin.

[ref-18] Comley J, Walker R, Wilson J, Ramsay A, Smith I, Raines P (2004). Malaysia coral reef conservation project: Pulau Redang.

[ref-19] Connell JH (1997). Disturbance and recovery of coral assemblages. Coral Reefs.

[ref-20] Croquer A, Pauls SM, Zubillaga AL (2003). White plague disease outbreak in a coral reef at Los Roques National Park, Venezuela. Revista De Biologia Tropical.

[ref-21] Croquer A, Weil E (2009). Changes in Caribbean coral disease prevalence after the 2005 bleaching event. Diseases of Aquatic Organisms.

[ref-22] Dalton SJ, Smith SDA (2006). Coral disease dynamics at a subtropical location, Solitary Islands Marine Park, eastern Australia. Coral Reefs.

[ref-23] Dinsdale EA, Moosa MK, Soemodihardjo S, Soegiarto A, Romimohtarto K, Nontji A, Suharsono S (2002). Abundance of black-band disease on corals from one location on the Great Barrier Reef: a comparison with abundance in the Caribbean region.

[ref-24] Doney SC (2006). The dangers of ocean acidification. Scientific American.

[ref-25] Fabricius KE, Langdon C, Uthicke S, Humphrey C, Noonan S, De’ath G, Okazaki R, Muehllehner N, Glas MS, Lough JM (2011). Losers and winners in coral reefs acclimatized to elevated carbon dioxide concentrations. Nature Climate Change.

[ref-26] Francini-Filho RB, Moura RL, Thompson FL, Reis RM, Kaufman L, Kikuchi RKP, Leão ZMAN (2008). Diseases leading to accelerated decline of reef corals in the largest South Atlantic reef complex (Abrolhos Bank, eastern Brazil). Marine Pollution Bulletin.

[ref-27] Gardner TA, Cote IM, Gill JA, Grant A, Watkinson AR (2003). Long-term region-wide declines in Caribbean corals. Science.

[ref-28] Goldberg J, Wilkinson C, Wilkinson C (2004). Global threats to coral reefs: coral bleaching, global climate change, disease, predator plagues and invasive species. Status of coral reefs of the world: 2004.

[ref-29] Haapkyl AJ, Seymour AS, Trebilco J, Smith D (2007). Coral disease prevalence and coral health in the Wakatobi Marine Park, south-east Sulawesi, Indonesia. Journal of the Marine Biological Association of the UK.

[ref-30] Harvell D, Jordan-Dahlgren E, Merkel S, Rosenberg E, Raymundo L, Smith G, Weil E, Willis B (2007). Coral disease, environmental drivers, and the balance between coral and microbial associates. Oceanography.

[ref-31] Harvell CD, Kim K, Burkholder JM, Colwell RR, Epstein PR, Grimes DJ, Hofmass EE, Lipp EK, Osterhaus ADME, Overstreet RM, Porter JW, Smith GW, Vasta GR (1999). Emerging marine diseases—climate links and anthropogenic factors. Science.

[ref-32] Harvell CD, Mitchell CE, Ward JR, Altizer S, Dobson AP, Ostfeld RS, Samuel MD (2002). Climate warming and disease risks for terrestrial and marine biota. Science.

[ref-33] Heron SF, Willis BL, Skirving WJ, Eakin CM, Page CA, Miller IA (2010). Summer hot snaps and winter conditions: modelling white syndrome outbreaks on great barrier reef corals. PLoS ONE.

[ref-34] Hoegh-Guldberg O, Mumby PJ, Hooten AJ, Steneck RS, Greenfield P, Gomez E, Harvell CD, Sale PF, Edwards AJ, Caldeira K, Knowlton N, Eakin CM, Iglesias-Prierto R, Muthiga N, Bradbury RH, Dubi A, Hatziolos ME (2007). Coral reefs under rapid climate change and ocean acidification. Science.

[ref-35] Jakobsen F, Hartstein N, Frachisse J, Golingi T (2007). Sabah shoreline management plan (Borneo, Malaysia): ecosystems and pollution. Ocean & Coastal Management.

[ref-36] Kaczmarsky LT (2006). Coral disease dynamics in the central Philippines. Diseases of Aquatic Organisms.

[ref-37] Kaczmarsky LT, Draud M, Williams EH (2005). Is there a relationship between proximity to sewage effluent and the prevalence of coral disease. Caribbean Journal of Science.

[ref-38] Knowlton N (2001). The future of coral reefs. Proceedings of the National Academy of Sciences of the United States of America.

[ref-39] Koch R (1892). Ueber bakteriologische Forschung. Verh X Int Med Congr Berlin.

[ref-40] Lamb JB, True JD, Piromvaragorn S, Willis BL (2014). Scuba diving damage and intensity of tourist activities increases coral disease prevalence. Biological Conservation.

[ref-41] Lindop AAM, Hind EJ, Bythell JC (2009). The unknowns in coral disease identification: an experiment to assess consensus of opinion amongst experts.

[ref-42] Miller I (1996). Black band disease on the Great Barrier Reef. Coral Reefs.

[ref-43] Miller J, Muller E, Rogers C, Waara R, Atkinson A, Whelan KRT, Patterson M, Witcher B (2009). Coral disease following massive bleaching in 2005 causes 60% decline in coral cover on reefs in the US Virgin Islands. Coral Reefs.

[ref-44] Muller EM, Rogers CS, Spitzack AS, Van Woesik R (2008). Bleaching increases likelihood of disease on *Acropora palmata* (Lamarck) in Hawksnest Bay, St John, US Virgin Islands. Coral Reefs.

[ref-45] NOAA (2006). Coral reef “Degree Heating Weeks.” NOAA Coral reef watch.

[ref-46] Nyström M, Folke C, Moberg F (2000). Coral reef disturbance and resilience in a human-dominated environment. Trends in Ecology & Evolution.

[ref-47] Palmer CV, Mydlarz LD, Willis BL (2008). Evidence of an inflammatory-like response in non-normally pigmented tissues of two scleractinian corals. Proceedings of the Royal Society B: Biological Sciences.

[ref-48] Palmer CV, Roth MS, Gates RD (2009). Red fluorescent protein responsible for pigmentation in trematode-infected porites compressa tissues. Biological Bulletin.

[ref-49] Pratchett MS, Hoey AS, Wilson SK (2014). Reef degradation and the loss of critical ecosystem services provided by coral reef fishes. Current Opinions in Environmental Sustainability.

[ref-50] Raymundo LJ, Couch CS, Bruckner AW, Harvell CD, Work TM, Weil E, Woodley CM, Jordan-Dahlgren E, Willis BL, Sato Y, Aeby GS (2008). Coral disease handbook: guidelines for assessment, monitoring & management. Coral Reef Targeted Research & Capacity Building for Management Program.

[ref-51] Reshef L, Koren O, Loya Y, Zilber-Rosenberg I, Rosenberg E (2006). The coral probiotic hypothesis. Environmental Microbiology.

[ref-52] Richardson LL, Rosenberg E, Loya Y (2004). Black band disease. Coral health and disease.

[ref-53] Ritchie KB (2006). Regulation of microbial populations by coral surface mucus and mucus-associated bacteria. Marine Ecology-Progress Series.

[ref-54] Ruiz-Morenol D, Willis BL, Page CA, Weil E, Corquer A, Vargas-Angel B, Jordan-Garza AG, Jordan-Dahlgren E, Raymundo L, Harvell CD (2012). Global coral disease prevalence associated with sea temperature anomalies and local factors. Diseases of Aquatic Organisms.

[ref-55] Sakari M, Ting LS, Juong LY, Lim SK, Tahir R, Adnan FAF, Yi ALJ, Soon ZY, Hsia BS, Shah MD (2012). Urban effluent discharge into rivers: a forensic chemistry approach to evaluate the environmenta deterioration. World Applied Sciences Journal.

[ref-56] Sutherland KP, Porter JW, Torres C (2004). Disease and immunity in Caribbean and Indo-Pacific zooxanthellate corals. Marine Ecology-Progress Series.

[ref-57] Sutherland KP, Porter JW, Turner JW, Thomas BJ, Looney EE, Luna TP, Meyers MK, Futch JC, Lipp EK (2010). Human sewage identified as likely source of white pox disease of the threatened Caribbean elkhorn coral *Acropora palmata*. Environmental Microbiology.

[ref-58] Sweet MJ, Croquer A, Bythel J (2014). Experimental antibiotic treatment identifies potential pathogens of white band disease in the endangered Caribbean coral *Acropora cervicornis*. Proceedings of the Royal Society B: Biological Sciences.

[ref-59] Venables WN, Ripley BD (2002). Modern applied statistics with S.

[ref-60] Weil E, Rosenberg E, Loya Y (2004). Coral disease in the wider Caribbean. Coral health and disease.

[ref-61] Wilkinson C (2008). Status of coral reefs of the world: 2008.

[ref-62] Wilkinson C, Souter D, Goldberg J (2006). Status of coral reefs in tsunami affected countries: 2005.

[ref-63] Willis BL, Page CA, Dinsdale EA, Rosenberg E, Loya Y (2004). Coral disease on the Great Barrier Reef. Coral health and disease.

[ref-64] Wood EW (2001a). SIDP Action Plan & Regulations for Tun Sakaran Marine Park. Draft action plan.

[ref-65] Wood EW (2001b). Conservation management issues. Semporna Islands Darwin project management plan.

[ref-66] project TRaR, WRI (2010). Status of coral reefs in Southeast Asia. Status of coral reefs.

[ref-67] Veron JEN, Stafford-Smith M (2000). Corals of the world.

[ref-68] Veron JEN, Hoegh-Guldberg O, Lenton TM, Lough JM, Obura DO, Pearce-Kelly P, Sheppard CRC, Spalding M, Stafford-Smith MG, Rogers AD (2009). The coral reef crisis: the critical importance of <350 ppm CO2. Marine Pollution Bulletin.

